# Artificial Production of the Flavors and Odors of Fruits and Flowers

**Published:** 1852-09

**Authors:** 


*Artificial Production of the Flavors and Odors of Fruits and Flowers.*—One of the most surprising achievements of modern chemistry is the artificial production of the flavors and odors of fruits and flowers; the imitation in the crude laboratory of the chemist of the most delicate of the productions of nature, and one which it might have been supposed was beyond the reach of art.

Dr. Playfair, in his lecture on the great exhibition of 1851, furnishes us with the following interesting information on this subject:—

"The jury in the exhibition, or rather two distinguished chemists of that jury, Dr. Hoffman and Mr. De la Rue, ascertained that some of the most delicate perfumes were made by chemical artifice, and not, as of old, by distilling them from flowers. The perfume of flowers often consists of oils and ethers, which the chemist can compound artificially in his laboratory. Commercial enterprise has availed itself of this fact, and sent to the exhibition, in the form of essences, perfumes thus prepared. Singularly enough, they are generally derived from substances of intensely disgusting odor. A peculiarly fetid oil, termed 'fusel oil,' is formed in making brandy and whiskey. This fusel oil, distilled with sulphuric acid, and acetate of potash, gives the oil of pears. The oil of apples is made from the same fusel oil, by distillation with sulphuric acid and bichromate of potash. The oil of pine-apples is obtained from the product of the action of putrid cheese on sugar; or by making a soap with butter, and distilling it with alcohol and sulphuric acid, and is now largely employed in England in the preparation of pine-apple ale. Oil of grapes, and oil of cognac, used to impart the flavor of French Cognac to British brandy, are little else than fusel oil. The artificial oil of bitter almonds, now so largely employed in perfuming soap, and for flavoring confectionery, is prepared by the action of nitric acid on the fetid oils of gas tar. Many a fair forehead is damped with eau de millefleurs, without knowing that its essential ingredient is derived from the drainage of cow-houses. The winter-green oil, imported from New Jersey, being produced from a plant indigenous there, is artificially made with willows and a body procured from the distillation of wood. All these are direct modern appliances of science to an industrial purpose, and imply an acquaintance with the highest investigations of organic chemistry. Let us recollect that the oil of lemons, turpentine, oil of juniper, oil of roses, oil of copaiba, oil of rosemary, and many other oils, are identical in composition, and it is not difficult to conceive that perfumery may derive still further aid from chemistry."—*Medical News.*

